# Multidimensional sleep health and resting blood pressure in young adults: the role of negative affect

**DOI:** 10.1007/s10865-026-00633-x

**Published:** 2026-02-04

**Authors:** Elizabeth J. Pantesco, Ethan J. Pastor, Ava N. Minchello, Diya A. Dudhia, Lila N. Nanagas

**Affiliations:** Department of Psychological and Brain Sciences, Villanova University, 800 Lancaster Avenue, Lancaster Avenue, Villanova, PA 19085 USA

**Keywords:** Sleep health, Blood pressure, Negative affect, Depression, Hostility

## Abstract

Poor sleep is increasingly recognized as a behavioral risk factor for cardiovascular disease. Few studies have examined how multidimensional sleep health and negative affect jointly relate to blood pressure (BP) in young adults. This study examined sleep health in relation to resting BP and tested whether negative affect accounted for or moderated the association. Participants were 129 young adults (mean age = 19.2 years, 70.5% female) without diagnosed sleep disorders. Sleep health was assessed using a composite index derived from actigraphy (regularity, timing, efficiency, duration) and self-report (satisfaction, alertness) metrics. Resting BP was measured in the laboratory. Hierarchical linear regression models were adjusted for demographics, body mass index, and attitudes toward sleep. Indicators of negative affect (depressive symptoms, anxiety, and hostility) were also included as covariates and tested as moderators. Better sleep health was associated with lower systolic BP only after adjusting for indicators of negative affect (B = − 1.40, SE = 0.63, *p* = .03). Hostility moderated associations between sleep health and systolic BP, such that effects were only significant among participants lower in hostility. These findings identify sleep health as a potential modifiable risk factor for hypertension and highlight the importance of considering both psychosocial context and multiple sleep dimensions when evaluating cardiovascular risk in early adulthood.

## Introduction

Poor sleep, characterized by short duration, low quality, irregular timing, and/or daytime consequences is increasingly recognized as a behavioral risk factor for cardiovascular disease (Makarem et al., 2022). As subclinical changes often precede the development of frank disease by years or even decades (Dalakoti et al., 2025; Ge et al., 2023), identifying pathways by which poor sleep contributes to cardiovascular risk earlier in the life course is critical. One proposed pathway is elevated blood pressure (BP), a major cardiovascular risk factor that is sensitive to behavioral and environmental influences. Although hypertension is often considered a condition of older adulthood, recent data show that nearly one in four young adults in the United States exhibits elevated BP (Fryar et al., 2024). Early identification of modifiable contributors such as sleep may offer opportunities for intervention before the onset of sustained hypertension, with implications for long-term cardiovascular risk reduction.

A growing body of evidence suggests that short sleep duration is associated with higher BP in children and adolescents (Jiang et al., 2018; Quist et al., 2016). However, as Sun et al. (2020) emphasize, much of this research has relied on self-reported sleep duration and has not accounted for the broader context of sleep health. Recent frameworks conceptualize sleep as a multidimensional construct that extends beyond duration to include parameters such as satisfaction, alertness, timing, efficiency, and regularity (Buysse, 2014; Micoulaud-Franchi et al., 2020). Composite sleep health indices, which combine various dimensions into a composite variable, provide a more comprehensive assessment of overall sleep functioning and may be useful in detecting associations between sleep and other indicators of health and well-being (Clementi et al., 2023; Griggs et al., 2023, 2024; İpar et al., 2025; Dong et al., 2019; Zarchev et al., 2025). Given that self-reported sleep measures may be prone to bias or recall error, composite indices that incorporate objective tools like actigraphy may offer a more accurate assessment of naturalistic sleep behavior on a prospective basis (Walton et al., 2025). While individual aspects of sleep health, such as duration (Cespedes Feliciano et al., 2018), efficiency (Javaheri et al., 2008), timing (Chang & Kang, 2021), and regularity (Culver et al., 2022), have each been linked to BP in adolescent and young adult samples, we are not aware of any studies examining a multidimensional composite measure of sleep in relation to BP in this age group.

Sleep health is closely linked with mental health and well-being, with the bidirectional associations between sleep and negative affect well-established. Poor or insufficient sleep increases risk for depressive symptoms (Marino et al., 2021), anxiety (Roberts & Duong, 2017), and anger or hostility (Van Veen et al., 2021; Zhang & Lei, 2023). These same factors, in turn, may disrupt sleep duration, quality, and timing. Notably, each of these indicators of negative affect has also been independently associated with elevated BP (Cuevas et al., 2017), although the direction of these associations is not always consistent across studies (Jeon et al., 2020). Thus, to more precisely estimate the unique association between sleep health and BP, it is critical to account for overlapping psychosocial factors (Hall et al., 2018; Mezick et al., 2011). Examining these factors together may also help determine whether young adults with elevated negative affect are especially vulnerable to the physiological consequences of poor sleep.

The aim of the current study was to examine the association between a composite sleep health measure and BP in a sample of healthy young adults. We hypothesized that better sleep health would be associated with lower BP and that these associations would be independent of negative affect. We also explored whether indicators of negative affect might moderate the association between sleep health and BP.

## Method

### Participants

Undergraduate students enrolled at a private university in the northeastern United States were recruited through the psychology department’s research participation system, online advertising, and campus flyers. Eligibility criteria included 18 years of age or older and English-speaking. Of the 135 participants who completed the study, we excluded five for reporting they had been diagnosed with a sleep disorder and one for not reporting their race and ethnicity, resulting in a final sample size of 129 participants.

### Measures

*Sleep Health Composite* A sleep health composite variable was calculated in line with previous work in adolescent and young adult samples (Clementi et al., 2023; Dong et al., 2019; Griggs et al., 2023, 2024; İpar et al., 2025; Zarchev et al., 2025), using a combination of actigraphy and self-report metrics. We followed the Ru-SATED framework, which proposes that regularity, satisfaction, alertness, timing, efficiency, and duration are key components of sleep health (Buysse, 2014). Criteria for healthy versus unhealthy standards were taken from previous work in similar age groups and/or expert recommendations when available (Clementi et al., 2023; Dong et al., 2019; Griggs et al., 2023, 2024; Hirshkowitz et al., 2015; İpar et al., 2025; Watson et al., 2015). In alignment with prior research, components meeting the healthy standard (described below) were awarded a point of 1, while those not meeting the standard received a score of 0. Scores were then summed into one composite variable, yielding a possible range from 0 to 6, with higher scores indicating better overall sleep health.

*Actigraphy Sleep Health Metrics* Sleep was assessed using the MotionWatch-8 (https://camntech.com), worn on the non-dominant wrist for five consecutive nights (three of which were weeknights). Participants were also asked to use the event marker button on the watch face and morning sleep diaries to indicate when they attempted to fall asleep and when they finally woke. Data were processed using CamNtech MotionWare v1.3.33 software, using a combination of the auto-sleep algorithm, diary reports, event marker data, and light levels. The four actigraphy variables used in analysis were *regularity*,* timing*,* efficiency*, and *duration*, as described below.

*Regularity* refers to the consistency of sleep timing, measured by the standard deviation of the actigraphy-assessed central phase (the midpoint between sleep onset and wake-up time, expressed in minutes past midnight). Participants with a standard deviation ≤ 1 h across the study period were given a score of 1, while those with greater irregularity were scored as 0.

*Timing* was based on the average actigraphy-assessed central phase across the study period. A midpoint between 2:00 and 4:00 AM was scored as 1 while a midpoint outside of that window was scored as 0.

*Efficiency* refers to the average percentage of time in bed asleep, as measured by actigraphy. An efficiency of 85% or higher was scored as 1; lower values were scored as 0.

*Duration* was assessed using actigraphy-assessed actual sleep time (total time scored as sleep based on epoch-by-epoch wake/sleep classification). Participants averaging 7 h or more of sleep per night received a score of 1, while those sleeping less received a score of 0.

*Self-Report Sleep Health Metrics* The Pittsburgh Sleep Quality Index (PSQI; Buysse et al., 1989) is a widely used self-report questionnaire assessing global sleep quality over the past month. We used several items from the PSQI to define the sleep health components of satisfaction and alertness, as described below.

*Satisfaction* was assessed using item 6 of the PSQI, which asks about quality of sleep over the past month. Reports of sleep quality as “fairly good” or “very good” were scored as 1, while reports of “fairly bad” or “very bad” were scored as 0.

*Alertness* was indexed using the daytime dysfunction subscale (Component 7) of the PSQI, which assesses difficulty staying awake and maintaining enthusiasm to complete tasks. Specifically, item 8 asks “During the past month, how often have you had trouble staying awake while driving, eating meals, or engaging in social activity?” and item 9 asks “During the past month, how much of a problem has it been for you to keep up enough enthusiasm to get things done?” Each of these two items are scored from 0 to 3, with higher scores reflecting greater daytime dysfunction. The two items are summed (range 0–6) and then categorized as follows: 0 = 0, 1–2 = 1, 3–4 = 2, 5–6 = 3. For use in the sleep health composite index, we recoded scores of 0 or 1 as 1 to indicate healthy alertness, while scores of 2 or 3 were recoded as 0.

*Blood Pressure* Resting BP was measured using a GE Carescape automated monitor while participants were seated quietly with feet flat on the floor. Cuff size was adjusted for arm circumference when needed. Two readings were taken approximately one minute apart with no experimenter interaction, and values were averaged to derive systolic (SBP) and diastolic (DBP) values.

*Depressive Symptoms* The Center for Epidemiological Studies Depression Scale (CES-D; Radloff, 1977) is a 20-item self-report measure of depressive symptomatology in the general population. Participants rated the frequency of their feelings and behaviors over the past week, with each response ranging from zero (“Rarely or none of the time”) to three (“Most or all the time”). A sample item is: “I thought my life had been a failure.” In typical use, total scores can range from 0 to 60, with higher scores indicating more severe depressive symptoms. In the current study, we removed the item asking about sleep disruption (CES-D item #11) in order to reduce confounding with sleep health scores, as done in prior work (Bowman et al., 2021; Lu et al., 2019). Internal consistency was high at α = 0.91.

*Anxiety* Anxiety was assessed using The Generalized Anxiety Disorder-7 (GAD-7; Spitzer et al., 2006). The GAD-7 was designed to assess generalized anxiety disorder symptoms within the past two weeks. Each item is self-reported according to the following range of responses: “not at all,” “several days,” “more than half the days,” and “nearly every day,” scored as 0, 1, 2 and 3 respectively. Sample items include: “feeling nervous, anxious, or on edge,” “trouble relaxing,” and “becoming easily annoyed or irritable”. Total score range is between 0 and 21, with higher scores reflecting higher levels of anxiety. Internal consistency for the GAD-7 in this sample was high (α = 0.91).

*Hostility* Hostility was assessed using the 27-item version of the Cook–Medley Hostility Scale (CMHS), an empirically derived subset of items from the original 50-item scale. This abbreviated version focuses on the core hostility dimensions of cynicism, hostile affect, and aggressive responding, which have been shown to be most predictive of cardiovascular outcomes (Barefoot et al., 1989). For each item, respondents are asked to indicate if the statement is “true” or “false,” with a sample item being “I think most people would lie to get ahead.” Each item is scored as either 0 or 1, with statements reflecting higher hostility scored as 1. Scores across all items are summed, with higher scores indicating higher levels of hostility. The 27-item CMHS has been widely used in psychosocial and health research to assess trait hostility in relation to cardiovascular risk (Steinberg & Jorgensen, 1996). The scale showed good internal consistency in the current sample (α = 0.79).

*Attitude Toward Sleep* The Charlotte Attitudes Toward Sleep (CATS; Peach & Gaultney, 2017) scale is a bidimensional 10-item measurement tool for assessing normal, non-dysfunctional sleep attitudes in two dimensions of sleep—sleep benefit/enjoyment and sleep as a time commitment—in daily life. Sleep benefit/enjoyment refers to positive attitudes towards sleep, with an example item being “I value getting a good night’s sleep”. Sleep as a time commitment is measured with items such as “I wake up early so I have more time to do other things.” Participants were presented with different phrases and asked to reply using a Likert type scale ranging from 1 (strongly disagree) to 7 (strongly agree). Items that were worded in a way to convey a negative attitude were reverse coded. Total higher scores indicate a more positive attitude towards sleep. Internal consistency in the current sample was strong (α = 0.82). We included the CATS scale as a covariate to account for sleep-specific cognitions that may overlap with negative affect and/or influence the subjective components of the composite sleep index (e.g., satisfaction and daytime sleepiness).

*Body Mass Index* Height and weight were measured using a calibrated mechanical column scale with an integrated stadiometer (Seca 700, Hamburg, Germany). Body mass index (BMI) was calculated as weight in kilograms divided by height in meters squared (kg/m²). BMI was included as a covariate given its links with sleep health and BP.

*Demographics* Participants self-reported their age, gender identity, race, and ethnicity (Hispanic versus non-Hispanic). They also reported their family socioeconomic status using the MacArthur Scale of Subjective Social Status (Adler et al., 2000). This scale presents an image of a 10-rung ladder meant to represent social standing across a number of status indicators (money, education, and job respect). Participants were asked to mark off the ladder rung that best represented their family’s status relative to others in the United States. Placement was scored continuously from 1 to 10, with 10 being the highest perceived status.

## Procedure

Participants provided informed consent and completed the PSQI, CATS scale, and demographic questionnaires at a baseline laboratory session. During this visit, they were fitted with an actigraph and had two resting BP measurements taken. Participants were instructed to wear the actigraph for the next five days and nights and to fill out a brief electronic sleep diary each morning. They also completed the CES-D, GAD, and CMHS before returning the actigraph. All study procedures were approved by the university’s ethics board.

### Analysis

After inspecting data for normality, we examined properties of the sleep health composite variable and bivariate correlations between main study variables. We then performed a series of hierarchical linear regression analyses predicting resting BP values from the sleep health composite variable, with separate models used for SBP and DBP. Sleep health was entered in the first step of the model, followed by demographic factors (age, gender, race and ethnicity, family social status), attitude toward sleep, and BMI in the second step. In the final step of the model, we entered depressive symptoms, anxiety, and hostility to determine their impact on the association between sleep health and BP. We then conducted exploratory analyses testing each indicator of negative affect as a moderator of the association between sleep health and BP by entering an interaction term (e.g., anxiety X sleep health). Significant interactions were probed using simple slopes analysis at high (84th percentile), moderate (median), and low (16th percentile) levels of the negative affect indicator (Hayes, 2022). Finally, we explored which dimensions of sleep health were most strongly related to BP by entering individual components into separate regression models adjusted for all covariates. We first tested each dimension in its categorical form (0 vs. 1), consistent with the manner in which dimensions were used in the sleep health composite variable. We then conducted a sensitivity analysis testing sleep regularity, timing, efficiency, and duration as continuous predictors of BP. These models were fully adjusted for all covariates listed above. All analyses were conducted using SPSS v.29, and statistical significance was set at *p* < .05.

## Results

**Fig. 1:**
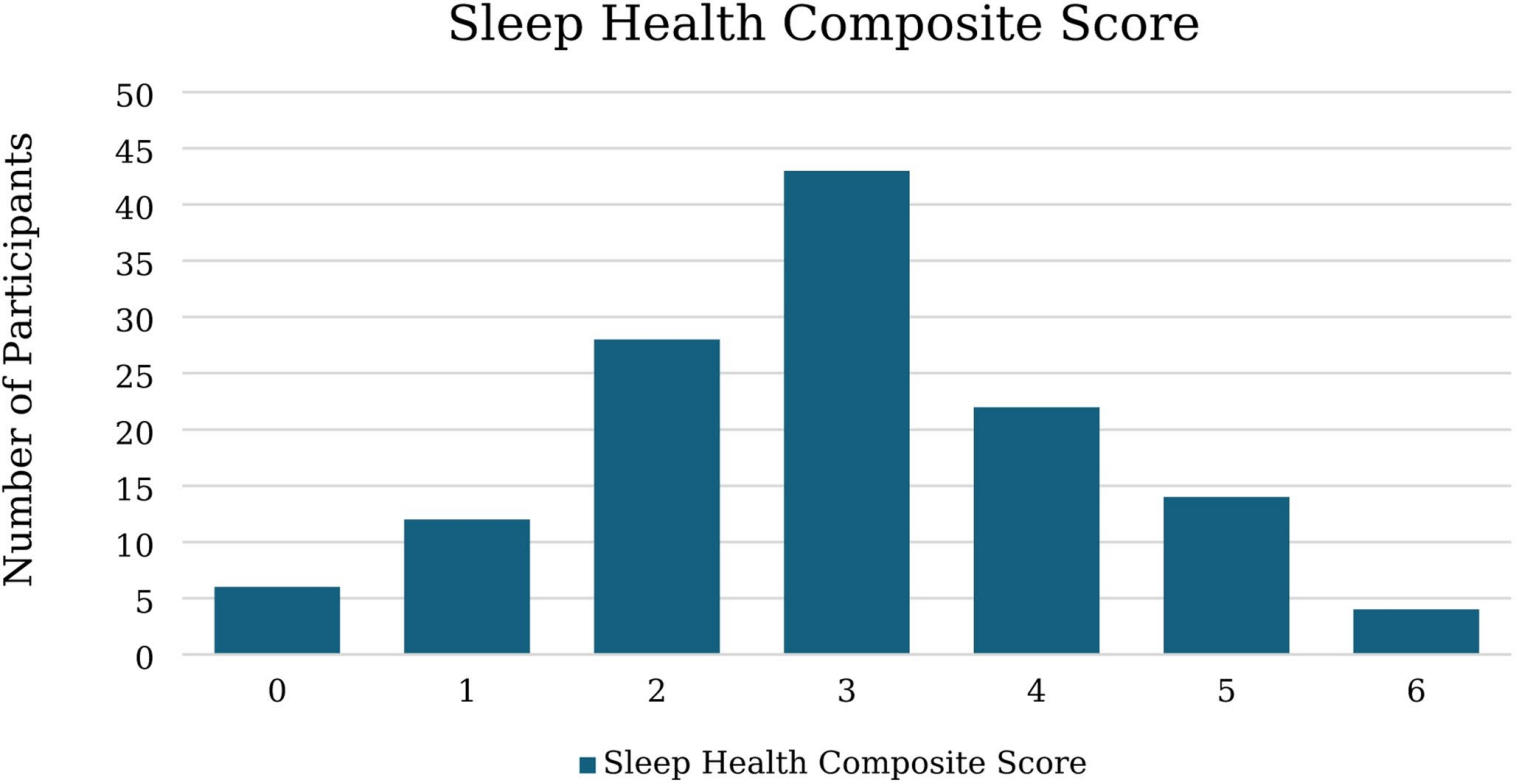
Distribution of multidimensional sleep health composite scores in full sample (*N* = 129)

Sample characteristics are shown in Table 1. The sample had a mean age of 19.18 (*SD* = 1.08) years and was largely female (70.5%), with just over half (55.8%) identifying as non-Hispanic White. The mean BMI was in the healthy range, as were SBP and DBP. Sleep health characteristics are presented in Table 2. Composite sleep health scores ranged from 0 to 6, with a mean of 2.94 (see Fig. 1). Internal consistency was low (KR-20/Cronbach’s α = 0.407), inter-item correlations averaged 0.106 (range: 0.049–0.364), and corrected item-total correlations averaged 0.199 (range: 0.043–0.293). Of the individual sleep dimensions, sleep satisfaction was the most frequently met criterion (75.2% of participants met the healthy threshold), while sleep timing was the least commonly met (15.5% of participants met the healthy criterion). Just over one-third of participants met the recommended sleep duration of at least 7 h (mean = 6.69 h, SD = 0.85). Bivariate correlations between sleep health, negative affect, and BP are shown in Table 3. Sleep health scores were negatively correlated with each indicator of negative affect (*p*s ≤ 0.01).

**Table 1:** Sample characteristics

	*N* (%)	Mean (SD)
Female	91 (70.5)	
Race or ethnicity		
Hispanic white	22 (17.1)	
Non-Hispanic white	72 (55.8)	
Other^a^	35 (27.1)	
Age		19.18 (1.08)
Body mass index		22.85 (3.81)
Family social status		4.43 (1.45)
Attitude toward sleep (CATS)		5.02 (0.58)
Depressive symptoms (CES-D^b^)		13.60 (9.46)
Anxiety (GAD-7)		7.26 (5.16)
Hostility (CMHS)		11.92 (4.83)
Systolic BP		112.63 (10.02)
Diastolic BP		63.83 (6.20)

CATS, Charlotte Attitude Towards Sleep Scale; CES-D, Center for Epidemiological Studies Depression Scale; GAD-7, Generalized Anxiety Disorder-7; CMHS, Cook-Medley Hostility Scale

^a^The “other” category was used for analytic purposes only and consisted of 11 Black or African American participants, 17 Asian participants, and 7 participants who endorsed 2 or more racial or ethnic categories

^b^with item #11 (disrupted sleep) removed

**Table 2:** Sleep health characteristics

	*N* (%)^a^	Mean (SD)	Range
Regularity (SD minutes)	71 (55.0)	61.55 (29.01)	12.70–145.01
Satisfaction	97 (75.2)		
Alertness	90 (69.7)		
Timing (minutes past midnight)	20 (15.5)	302.63	158.10–501.55
Efficiency (%)	57 (44.2)	83.86	65.10–92.92
Duration (hours)	44 (34.1)	6.69	4.01–9.18

^a^N (%) indicates number (percent) of sample that met the healthy criterion

**Table 3:** Correlations between main study variables

	1.	2.	3.	4.	5.
1. Sleep health composite score	–	–	–	–	–
2. Depressive symptoms (CES-D)	− 0.43^**^	–	–	–	–
3. Anxiety (GAD-7)	− 0.34^**^	0.71^**^	–	–	–
4. Hostility (CMHS)	− 0.23^*^	0.52^**^	0.37^**^	–	–
5. Systolic blood pressure	− 0.11	− 0.12	− 0.14	− 0.07	–
6. Diastolic blood pressure	− 0.03	− 0.06	− 0.002	− 0.10	0.38^**^

CES-D, Center for Epidemiological Studies Depression Scale; GAD-7, Generalized Anxiety Disorder-7; CMHS, Cook-Medley Hostility Scale

*p < .01, **p < .001

Results from linear regression models are presented in Table 4. After adjusting for demographics, attitude toward sleep, and BMI, sleep health was not associated with SBP (β = − 0.13, *p* = .11). After adding depressive symptoms, anxiety symptoms, and hostility in the second step of the model, better sleep health was associated with lower SBP (β = − 0.19, *p* = .03). The inverse association between depressive symptoms and SBP approached but did not reach significance. The final model accounted for 31.0% of the variance in SBP (sleep health R^2^∆ = 0.03). In terms of DBP, sleep health scores were not significantly associated with DBP before (β = − 0.06, *p* = .54) or after (β = − 0.08, *p* = .41) adjusting for indicators of negative affect. The final model accounted for 2.1% of the variance in DBP (sleep health R^2^∆ = 0.003).

**Table 4:** Effects from linear regression models of sleep health, negative affect, and blood pressure

Systolic blood pressure					Diastolic blood pressure		
		B (SE)	95% CI	*p*	B (SE)	95% CI	*p*
Sleep health^a^		− 0.97 (0.59)	− 2.14, 0.21	0.11	− 0.27 (0.43)	− 1.12, 0.59	0.54
CES-D		− 0.23 (0.12)	− 0.48, 0.01	0.06	− 0.10 (0.09)	− 0.29, 0.08	0.25
GAD-7		0.04 (0.21)	− 0.38, 0.46	0.84	0.13 (0.16)	− 0.18, 0.44	0.40
CMHS		0.14 (0.19)	− 0.23, 0.52	0.45	− 0.01 (0.14)	− 0.29, 0.27	0.95
Sleep health^b^		− 1.40 (0.63)	− 2.63, − 0.16	0.03	− 0.38 (0.46)	− 1.29, 0.53	0.41
CES-D x Sleep health		0.01 (0.06)	− 0.10, 0.13	0.80	− 0.05 (0.04)	− 0.13, 0.03	0.26
GAD-7 x Sleep health		0.04 (0.12)	− 0.20, 0.27	0.76	− 0.11 (0.09)	− 0.28, 0.06	0.20
CMHS x Sleep health		0.22 (0.11)	0.01, 0.43	0.04	0.14 (0.08)	− 0.02, 0.29	0.08

CES-D, Center for Epidemiological Studies Depression Scale; GAD-7, Generalized Anxiety Disorder-7; CMHS, Cook-Medley Hostility Scale. Models are adjusted for age, gender, race and ethnicity, social status, attitude toward sleep, and BMI

^a^Effect for sleep health prior to entering negative affect indicators in model

^b^Effect for sleep health after entering negative affect indicators in model

**Fig. 2:**
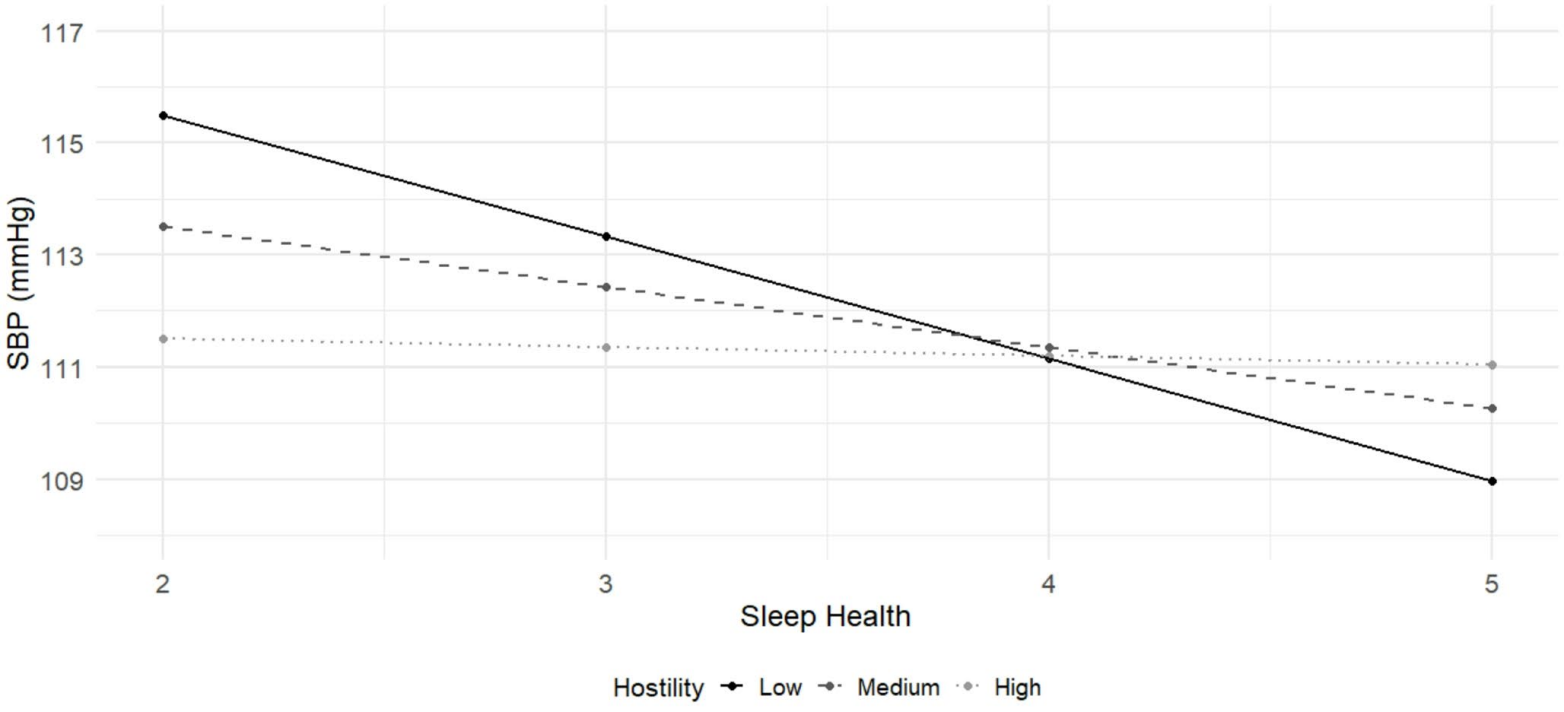
Associations between sleep health and systolic blood pressure at low, medium, and high levels of hostility

We next examined whether associations between sleep health and BP were conditional upon indicators of negative affect. Results are presented in Table 4. Neither depressive symptoms nor anxiety moderated the associations between sleep health and BP. Hostility was a significant moderator of the association between sleep health and SBP (β = 0.46, *p* = .04, R^2^∆ = 0.03). Simple slopes analysis showed that the association between sleep health and SBP was significant only at lower levels of hostility (Table 5; Fig. 2). The Johnson–Neyman technique identified a transition point at a hostility score of 11.50, above which the effect of sleep health on SBP was nonsignificant. Hostility was not a significant moderator of the relationship between sleep health and DBP (β = 0.47, *p* = .08, R^2^∆ = 0.02).

**Table 5:** Associations between sleep health and systolic blood pressure at low, medium, and high hostility

	B (SE)	95% CI	*p*
Low CMHS	− 2.18 (0.83)	− 3.81, − 0.54	0.01
Medium CMHS	− 1.08 (0.59)	− 2.25, 0.10	0.07
High CMHS	− 0.15 (0.72)	− 1.57, 1.27	0.83

CMHS, Cook-Medley Hostility Scale. Low = 16th percentile, Medium = 50th percentile; High = 84th percentile

*Individual Components of Sleep Health* We entered each component of sleep health as a predictor of BP in a linear regression model. Results are presented in Supplementary Tables 1 and 2. After adjustment for all covariates, increased daytime alertness (measured dichotomously; β = − 0.19, *p* = .04, R^2^∆ = 0.03) and higher sleep efficiency (measured continuously; β = − 0.16, *p* = .04, R^2^∆ = 0.02) were both associated with lower SBP. None of the other individual sleep health components were significantly associated with SBP or DBP.

## Discussion

In this study of healthy young adults, better multidimensional sleep health was associated with lower resting systolic blood pressure (SBP) after adjusting for negative affect, attitude toward sleep, BMI, and demographic factors. This association was moderated by hostility, such that the relationship between sleep health and SBP was only significant among those lower in hostility. There was no association between sleep health and diastolic blood pressure (DBP). Overall, these findings underscore the relevance of multidimensional sleep health for cardiovascular risk profiles in emerging adults and support the importance of considering psychosocial factors when evaluating sleep–BP associations.

The mean sleep health score of 2.94 in the current study is similar to index scores reported in recent studies that incorporated both actigraphy and self-reported sleep dimensions in young adult or older adolescent samples (Clementi et al., 2023; Nyhuis et al., 2023; Zarchev et al., 2025). Both Nyhuis et al. (2023) and Clementi et al. (2023) observed similarly low rates of participants (25% and 35%, respectively) meeting healthy sleep duration cut-offs, consistent with the prevalence of inadequate sleep observed in epidemiological studies of young adults (Pankowska, 2023). However, very few participants in the current study met the healthy sleep midpoint timing of 2–4 am. This is most likely due to our focus on a university sample, in which the combination of circadian influences, academic and social demands, and increased technology use commonly result in later bedtimes (Yeom et al., 2024). While we opted to use a timing criterion consistent with prior work for comparison, future research could explore population-specific cutpoints to better capture normative sleep timing in university students.

Our finding that better sleep health was associated with lower systolic BP is consistent with recent studies linking multidimensional sleep health indices to improved physical and functional health outcomes, including lower diabetes symptom burden (Griggs et al., 2024), achievement of glycemic targets (Griggs et al., 2023), and reduced headache-related disability (Clementi et al., 2023), in adolescents and young adults. Moreover, studies in adults have reported relationships between sleep health and increased risk for cardiovascular outcomes, including hypertension (Makarem et al., 2022), heart disease in mid-life adults (Lee et al., 2022), and CVD mortality in older adults (Wallace et al., 2019). Notably, several of these studies found the composite index was a stronger predictor than individual sleep components, highlighting the value of adopting a multidimensional approach. Exploratory analysis in the current study showed that sleep efficiency and alertness were the two sleep health dimensions related to SBP, suggesting that sleep difficulties impacting daytime functioning may be especially relevant to cardiovascular risk in this age group. Future work replicating and refining sleep health models in relation to cardiovascular health may help inform the development of early risk profiles and targeted preventions strategies for young adults.

Given the overlap among psychological well-being, sleep, and cardiovascular risk, one of our aims was to investigate how psychological factors influence the relation between sleep health and BP. Sleep health was associated with SBP only after accounting for indicators of negative affect. This likely reflects a suppressor effect, as depressive symptoms were linked to poorer sleep but lower SBP in this sample, suggesting that sleep health and depressive symptoms may relate to BP regulation through distinct behavioral or physiological mechanisms. Similar inverse associations of BP with depressive symptoms and/or anxiety have been reported, though findings across cohorts remain mixed (Hildrum et al., 2011; Jeon et al., 2020; Schaare et al., 2023). For example, recent work using UK Biobank data demonstrated that higher SBP was associated with better mood and fewer depressive symptoms among normotensive individuals, leading the authors to propose a transient regulatory benefit of elevated BP in which negative affect is blunted through baroreceptor-mediated attenuation of emotional reactivity (Schaare et al., 2023). While speculative, our findings align with this view: in a healthy, young adult sample, depression may suppress the expected association between poor sleep and higher BP, possibly via distinct central autonomic or baroreflex pathways. This effect underscores the need for longitudinal research to clarify how depressive symptoms and sleep health jointly shape BP regulation across development.

We observed that hostility moderated the sleep-BP relationship, such that sleep health was related to lower SBP at low, but not high, hostility levels. Notably, the point at which the association between sleep health and SBP became nonsignificant (Cook–Medley hostility score ≈ 11.5) corresponds closely to the score previously proposed to indicate clinically meaningful hostility (Barefoot et al., 1989). Although we anticipated a cumulative risk model in which the combination of high hostility and poor sleep was associated with elevated BP, these findings suggest that the association between healthier sleep and lower BP may be most evident among individuals low in interpersonal negativity. One possibility is that poor sleep represents a relatively novel or destabilizing stressor for those low in hostility, producing greater physiological reactivity. In contrast, baseline stress-regulatory systems may be more consistently engaged among those higher in hostility, making subtle improvements in sleep less influential. Alternatively, low hostility may index healthier behaviors (Wong et al., 2013), such as regular exercise, a balanced diet, or reduced alcohol consumption, that amplify the cardiovascular benefits of good sleep. Overall, these data suggest that the cardiovascular relevance of sleep health may depend on broader psychosocial context. Routinely assessing sleep health alongside psychosocial factors may be particularly valuable when evaluating cardiovascular risk in young adults, for whom risk processes are still emerging and behavioral interventions may be most feasible. Future research may wish to examine whether interventions targeting sleep health confer differential cardiovascular benefits depending on personality traits such as hostility.

Experimental studies suggest that poor sleep health may causally influence BP through mechanisms such as autonomic dysregulation, endothelial dysfunction, and systemic inflammation (Hall et al., 2018). In addition, sleep extension protocols have been shown to reduce BP (Mathew et al., 2024), especially when sleep efficiency also improves (Gonzales et al., 2024). While our findings emphasize the potential impact of sleep health on BP, the cross-sectional study design prevents conclusions about causal ordering among variables. Poor sleep may contribute to negative affect, affective symptoms may disrupt sleep, and BP may both influence and reflect these processes. The present findings therefore characterize conditional associations rather than temporal pathways. Longitudinal and experimental studies that repeatedly assess sleep, affect, and cardiovascular function will be essential for clarifying directionality and identifying points of intervention. Other limitations of the current study include the young, healthy sample, which limits generalizability and reduces BP variability, potentially obscuring stronger associations that could emerge in higher-risk or more heterogeneous populations. Moreover, the majority of participants were female, and previous work has indicated that links between multidimensional sleep health and cardiovascular risk may vary by gender (Makarem et al., 2022). While the sample had a fair degree of racial/ethnic diversity, we were inadequately powered to examine variation in associations by race. We did not measuremedication use or napping, both of which will be important to assess in future work aiming to understand sleep and BP especially in the context of depressive symptoms. Finally, we relied on resting BP measurements assessed during a single session, and future studies could consider inclusion of ambulatory BP monitoring given its stronger predictive validity and ability to capture circadian BP patterns (Eguchi et al., 2008).

In summary, multidimensional sleep health is linked to lower SBP in healthy young adults, with daytime alertness and sleep efficiency emerging as relevant components. The association emerged after adjusting for negative affect and was only present among individuals with lower levels of hostility, highlighting the importance of considering both behavioral and psychosocial pathways in early cardiovascular risk. These results suggest that promoting good sleep health early in adulthood may represent a promising avenue for mitigating early cardiovascular risk. However, longitudinal research is needed to clarify causal pathways and examine whether improvements in sleep confer sustained benefits for BP across development.

## Data Availability

The data that support the findings of this study are not publicly available due to participant privacy restrictions and institutional review board requirements. De-identified data may be made available from the corresponding author upon reasonable request and with institutional approval.
